# Some like it hot: Repeat migration and residency of whale sharks within an extreme natural environment

**DOI:** 10.1371/journal.pone.0185360

**Published:** 2017-09-21

**Authors:** David P. Robinson, Mohammed Y. Jaidah, Steffen S. Bach, Christoph A. Rohner, Rima W. Jabado, Rupert Ormond, Simon J. Pierce

**Affiliations:** 1 Heriot-Watt University, Edinburgh, United Kingdom; 2 Qatar Ministry of Environment, Doha, Qatar; 3 North Oil Company, Doha, Qatar; 4 Marine Megafauna Foundation, Truckee, CA, United States of America; 5 Gulf Elasmo Project, Dubai, United Arab Emirates; 6 Marine Conservation International, Edinburgh, United Kingdom; University of California Davis, UNITED STATES

## Abstract

The Arabian Gulf is the warmest sea in the world and is host to a globally significant population of the whale shark *Rhincodon typus*. To investigate regional whale shark behaviour and movements, 59 satellite-linked tags were deployed on whale sharks in the Al Shaheen area off Qatar from 2011–14. Four different models of tag were used throughout the study, each model able to collect differing data or quantities of data. Retention varied from one to 227 days. While all tagged sharks crossed international maritime boundaries, they typically stayed within the Arabian Gulf. Only nine sharks dispersed through the narrow Strait of Hormuz into the Gulf of Oman. Most sharks stayed close to known or suspected feeding aggregation sites over summer months, but dispersed throughout the Arabian Gulf in winter. Sharks rarely ventured into shallow areas (<40 m depth). A single, presumably pregnant female shark was the sole animal to disperse a long distance, crossing five international maritime boundaries in 37 days before the tag detached at a distance of approximately 2644 km from the tagging site, close to the Yemeni-Somali border. No clear space-use differentiation was evident between years, for sharks of different sizes, or between sexes. Whale sharks spent the most time (~66%) in temperatures of 24–30°C and in shallow waters <100 m depth (~60%). Sharks spent relatively more time in cooler (X^2^ = 121.692; p<0.05) and deeper (X^2^ = 46.402; p<0.05) water at night. Sharks rarely made dives deeper than 100 m, reflecting the bathymetric constraints of the Gulf environment. Kernel density analysis demonstrated that the tagging site at Al Shaheen was the regional hotspot for these sharks, and revealed a probable secondary aggregation site for whale sharks in nearby Saudi Arabian waters. Analysis of visual re-sightings data of tagged sharks revealed that 58% of tagged individuals were re-sighted back in Al Shaheen over the course of this study, with 40% recorded back at Al Shaheen in the year following their initial identification. Two sharks were confirmed to return to Al Shaheen in each of the five years of study.

## Introduction

The world’s largest fish, the whale shark, *Rhincodon typus* (Smith, 1828), has routinely been described as enigmatic [1,2], as aspects of its biology and habitat use remain poorly understood. For example, knowledge of the species’ reproduction is lacking [2,3] and encounters with neonates are a rare occurrence [3,4]. Whale sharks routinely move across international boundaries and political jurisdictions [5–9]. However, whale sharks can show a significant degree of site fidelity. Berumen et al. [5] tagged 47 sharks in the southern Red Sea for periods of 11 to 315 days, with only eight sharks swimming farther than ~800 km from the tagging location. Passive acoustic tagging studies off Mafia Island in Tanzania demonstrated high whale shark residency to a small embayment for periods of up to two years [10].

Rowat & Brooks [3] reported that, although whales sharks tagged with satellite-linked tags at aggregation sites have been shown to make long-distance movements, a subsequent return migration to the tagging site had not been demonstrated. Whale sharks occur and aggregate with predictable timing at a number of specific locations around the world [11]. Prey availability is thought to be the primary driver behind the movements of whale sharks and their arrival at aggregation sites [12,13]. However, it remains unclear whether movements and migrations are solely driven by prey availability or linked to other aspects of the whale shark’s life history [7].

Whale sharks are capable of dives into the bathypelagic zone. The deepest dive recorded to date was 1928 m from a whale shark tagged off the Yucatan Peninsula, Mexico, which was also the deepest documented dive from any fish [9]. The reasons for these deep diving excursions are not known, but could be related to feeding [14], navigation, or to reduce energy expenditure while travelling [15]. Fatty acid studies have found that deepwater zooplankton and mesopelagic fishes may comprise a portion of the diet [16,17]. However, while most whale shark tracking studies to date have recorded irregular deep dives to >1000 m, the sharks typically spent the majority of their time in the epipelagic zone [3]. Where sufficient prey is available, whale sharks have been observed to spend long periods in shallow water while foraging [7,18,19]. Whale sharks feeding off Holbox, Mexico spent 43% of time at the surface during the day compared to 16% at night [20] and whale sharks tagged in the Gulf of Mexico spent approximately 95% of their time in water depths <200m while in an oceanic environment [9].

Whale sharks are ectotherms and, there is evidence that whale sharks undertake behavioural thermoregulation [21]. After prolonged deep dives to water as cold as 3.4°C [22], they spend extended periods at the surface, possibly to warm up [21]. In the Arabian Gulf, however, they face the opposite challenge: water temperatures at the surface can be >35°C in summer, and they spend many hours in this surface layer feeding on tuna eggs [23]. Despite being shallow, with a maximum depth of just over 90 m [6], the Arabian Gulf has cooler water at depth, as low as 18°C, even in summer (S. Bach unpubl. data).

Most whale shark feeding aggregations are highly seasonal, and shark movements are generally poorly known outside these times, although large scale, multi-year movement studies have taken place at some sites around the world [5,7]. During the boreal summer the Al Shaheen area in Qatar hosts a large aggregation of whale sharks which feed there on freshly released tuna eggs [6,23].

Due to the whale sharks endangered status [24], known bycatch in surrounding areas [24], and susceptibility to injury from large vessel traffic [25], we aimed to investigate the movement ecology of the whale sharks that utilise the Al Shaheen area using satellite-linked tags. We investigate both the horizontal and vertical movements of these sharks in this hot, semi-enclosed, shallow environment. We used satellite tags to examine their diving behaviours, depth preferences, preferred temperature ranges and spatial habitat use, and integrate these data with concurrent photo-identification studies to investigate return migration to the Al Shaheen area.

## Materials and methods

### Whale sharks tagging area

**Fig 1:**
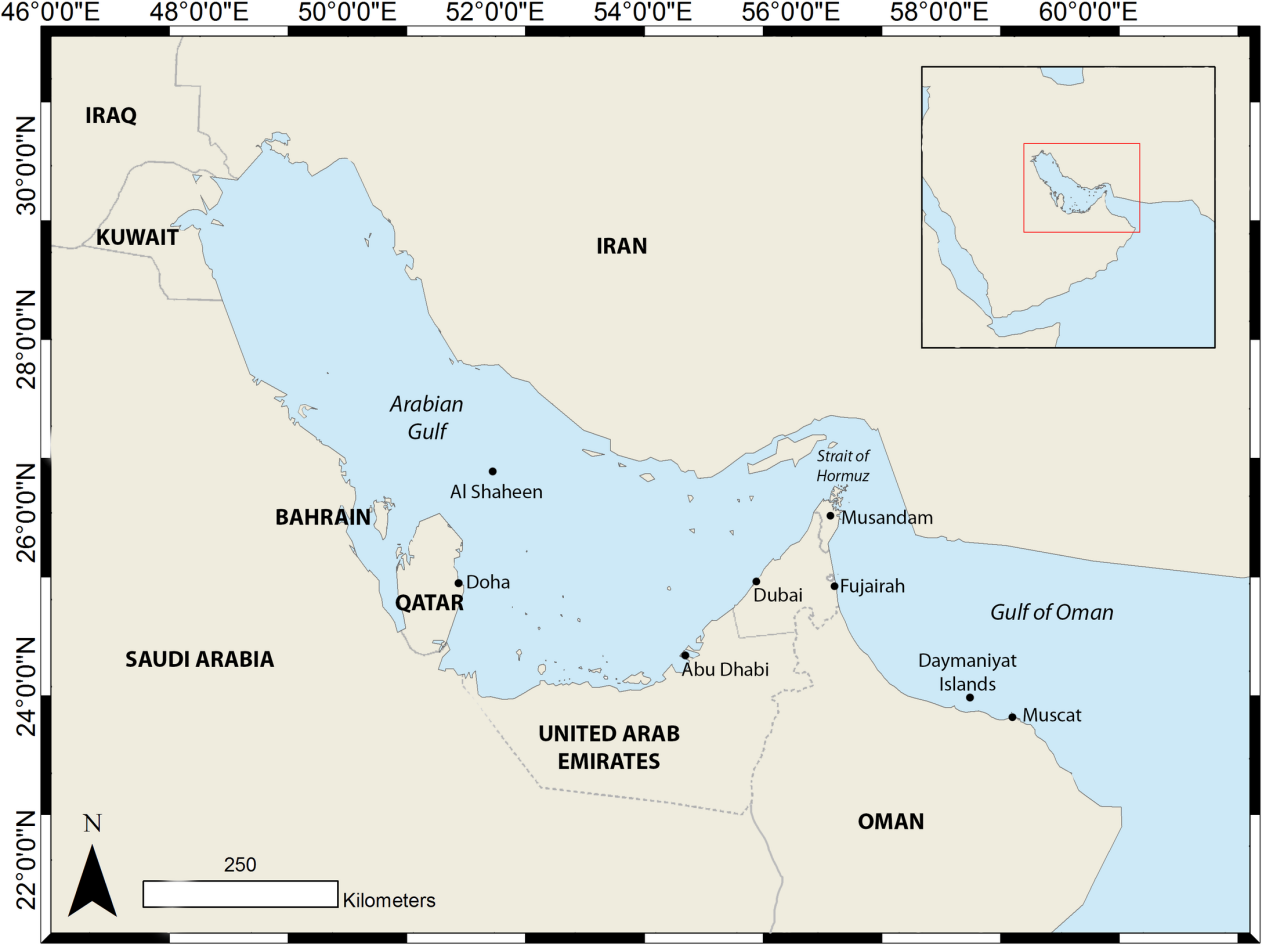
The locations of all study sites and other points of interest for whale sharks within the Arabian Gulf and Gulf of Oman (wider region shown in inset).

Whale sharks were tagged in Al Shaheen (Fig 1) between July 2011 and September 2014. Fifty-nine Wildlife Computers satellite tags were used, consisting of four different models: Pop-Off Archival Tags (PAT) model MK10 (n = 10) and MiniPAT tags (n = 10; Table 1); near-real-time SPOT5 tags (n = 28; Table 2) and ‘hybrid’ tags (PAT + real time) MK10F (n = 11; Table 3), henceforth referred to as towed tags. PAT tags recorded light levels, depth, and temperature, and were programmed to release from the shark after four, six, or 12 months. Towed tags recorded Argos locations when at the surface as well as temperature data.

**Table 1:** Summary data from 20 PAT tags deployed on male (M) and female (F) whale sharks in Al Shaheen, Qatar.

PTT ID	Tag Type	TL (m)	Sex	Wildbook Alternate ID	Date Deployed	Date Detached	Set for (days)	Data Collection (days)	Max Depth (m)	Decoded Data (%)	Max Temp (°C)	Min Temp (°C)
75731	MK10	8	F	qat11-001	23.04.11	02.07.11	180	73	72	67	32.2	19.2
75730	MK10	8	F	Unknown	14.05.11	11.09.11	120	120	80	69	35	19
75831	MK10	8	M	Unknown	14.05.11	No report	120	No report	No report	NA	No report	No report
75832	MK10	8	M	Unknown	14.05.11	11.09.11	120	120	72	55	35.4	19.8
75855	MK10	6	F	Unknown	14.05.11	No report	120	No report	No report	NA	No report	No report
104001	MK10	4	M	qat11-027	09.07.11	19.07.11	120	10	64	84	34	20.8
108547	MK10	5	M	qat11-026	09.07.11	20.07.11	120	11	64	91	34	20.4
110446	MK10	8	M	qat12-018	27.05.12	29.06.12	365	33	80	78	33.4	18.2
110447	MK10	7	F	qat12-063	27.05.12	30.08.12	365	104	8	45	No report	No report
103238	MK10	9	M	qat14-021	28.05.14	No report	180	No report	No report	NA	No report	No report
119147	MiniPAT	6	F	qat12-173	13.07.12	No report	365	No report	No report	NA	No report	No report
119148	MiniPAT	6	F	qat12-144	13.07.12	09.01.13	180	180	74	77	No report	No report
119149	MiniPAT	7	F	qat12-039	27.05.12	No report	365	No report	No report	NA	No report	No report
119150	MiniPAT	6	M	qat12-165	13.07.12	09.01.13	180	180	80	76	No report	No report
119151	MiniPAT	6	M	qat12-062	13.07.12	09.01.13	180	180	136	68	No report	No report
132228	MiniPAT	7	F	qat13-085	19.09.13	18.03.14	180	180	228	72	35	20.9
132229	MiniPAT	5	M	qat13-107	19.09.13	18.03.14	180	180	110	68	35.1	19.9
132230	MiniPAT	8	M	qat13-106	19.09.13	05.02.14	180	137	104	67	34	20.4
132231	MiniPAT	7	M	qat13-095	19.09.13	18.03.14	180	180	88	70	34	19.5
132232	MiniPAT	7	F	qat13-098	19.09.13	18.03.14	180	180	344	69	34.7	17.6
**Total Mean for Males**								**115**	**89**	**73**	**34**	**20**
**Total Mean for Females**								**140**	**134**	**67**	**34**	**19**
**Total Mean for both Male and Female**								**125**	**107**	**70**	**34**	**20**

**Table 2:** Summary of data from 28 SPOT5 tags deployed on male (M) and female (F) whale sharks in Al Shaheen, Qatar, with mean days of data collection and mean tracked distance for each.

PTT ID	TL (m)	Sex	Wildbook Alternate ID	Date Deployed	Date Detached	Data Collection (days)	Track Distance (km)	Mean Dist / day (km)
129051	7	F	qat12-180	29.06.13	22.08.13	54	10	0.19
129052	8	M	qat13-068	30.06.13	29.11.13	152	264	1.74
129053a	6	M	qat11-090	16.05.13	29.05.13	13	52	4.00
129054	7	M	qat11-062	01.07.13	02.07.13	1	0	NA
129055	8	M	qat13-040	01.07.13	16.08.13	47	671	14.28
132226	7	M	qat13-112	19.09.13	12.12.13	84	244	2.90
132227	5	M	mus12-010	19.09.13	24.01.14	127	919	7.24
120998	8	M	qat13-056	02.07.13	03.11.13	124	845	6.81
120999	9	M	qat13-032	02.07.13	26.07.13	24	32.6	1.36
128519	6	F	qat13-076	02.07.13	11.07.13	9	14	1.56
128520	7	F	qat13-090	12.09.13	07.10.13	25	212	8.48
121000	7	M	qat11-073	19.05.14	28.06.14	40	148	3.70
132221	7	F	qat13-102	19.05.14	01.06.14	12	20	1.67
132222	9	F	qat14-023	28.05.14	04.07.14	37	2613	70.62
132223	8	M	qat12-256	28.05.14	24.07.14	57	174	3.05
132224a	8	M	qat14-013	28.05.14	26.06.14	29	35	1.21
132225	8	M	qat12-045	27.06.14	09.02.15	227	564	2.48
129053b	9	M	qat14-037	27.06.14	29.06.14	2	0	NA
138518	7	F	qat12-173	27.06.14	09.09.14	74	192	2.59
138521	7	M	qat12-019	27.06.14	25.08.14	59	131	2.22
138517	7	F	qat14-042	15.08.14	09.11.14	86	120	1.40
138519	8	M	qat11-024	15.08.14	13.10.14	59	129	2.19
138520	7	F	qat13-090	15.08.14	12.10.14	58	245	4.22
132224b	5	F	qat14-046	15.08.14	20.03.15	217	618	2.85
141894	6	M	qat14-069	16.09.14	27.02.15	164	1158	7.06
141895	6	F	qat14-056	16.09.14	18.10.14	63	146	2.32
141896	5	M	qat12-062	16.09.14	09.10.14	23	154	6.70
141897	5	F	qat13-109	16.09.14	22.11.14	67	870	12.99
**Total Mean for Males**						**72**	**368**	**4**
**Total Mean for Females**						**64**	**460**	**10**
**Total Mean for both Male and Female**						**69**	**406**	**7**

**Table 3:** Summary of data from 11 MK10F tags deployed on male (M) and female (F) whale sharks in Al Shaheen, Qatar, with mean days of data collection and mean depth, maximum temperature, minimum temperature and tracked distance.

PTT ID	TL (m)	Sex	Wildbook Alternate ID	Date Deployed	Date Detached	Set for (days)	Data Collection (days)	Max Depth (m)	Max Temp (°C)	Min Temp (°C)	Track Distance (km)
119152	6	F	qat12-169	13.07.12	08.01.13	180	180	116	35.5	19	262
119153	8	M	qat11-019	27.05.12	23.11.12	180	180	88	35	18	376
119154	7	M	qat12-105	01.06.12	17.06.12	180	17	58	18.8	34	78
119155	6	F	qat11-018	01.06.12	01.06.12	180	No report	0	0	0	0
119156	7	F	qat12-078	01.06.12	01.06.12	180	No report	0	0	0	0
129822	8	M	qat12-093	30.06.13	15.09.13	180	67	80	35.8	21.5	314
129823	8	M	qat11-097	30.06.13	04.10.F13	180	96	80	36	21.7	177
138515	8	M	qat13-071	27.06.14	03.11.14	180	129	208	34.5	20	783
138516	6	M	qat14-041	15.08.14	08.10.14	180	54	74	35	21.3	134
137642	8	F	qat12-153	20.08.14	07.10.14	180	48	96	35.7	21	135
137643	7	M	qat14-067	16.09.14	05.11.14	180	50	208	34.1	21.7	587
**Total Mean for Males**							**85**	**114**	**33**	**23**	**350**
**Total Mean for Females**							**114**	**106**	**36**	**20**	**199**
**Total Mean for both Male and Female**							**91**	**112**	**33**	**22**	**316**

Permissions for fieldwork and data collection on whale sharks in the Al Shaheen region of Qatar were given by the Qatar Ministry of Environment with whom this work was conducted. The whale shark is listed as 'Endangered' on the IUCN Red List [24] but is not protected under law in Qatari waters where the fieldwork was carried out. All satellite tags were deployed while snorkeling alongside free-swimming whale sharks. Researchers took photographs of the flank area on the left side of the shark for individual identification [26]. Sex was determined through the presence or absence of claspers and, maturity in male sharks was determined by calcification of the claspers. Estimated length of individual sharks was recorded to the nearest metre [23]. Presumed pregnancy in female sharks was assessed using both estimated TL and the presence of a distinctive swollen abdomen as described in Acuña-Marrero et al [27]. Re-sightings of previously tagged individuals using their identification images within the Wildbook for Whale Sharks photo-identification library (http://www.whaleshark.org) allowed us to continue tracking the sharks’ movements after tag detachment and also ascertain their survival post-tagging [6].

A Wildlife Computers titanium anchor dart was used to anchor the tag within the shark. The dart was inserted into the dermal layer on the left dorsal side of the shark, directly below the centre line of the dorsal fin. A 6 ft pole spear was used to apply the tags in 2011 and 2012. A metal bush was designed to attach the Wildlife Computers applicator to the pole spear and rubber bungs were set at 10 cm depth to stop the applicator penetrating deeper into the shark. The pole spear could not penetrate the thick skin of large sharks of >8 m total length (TL), and was replaced with a pneumatic spear gun in 2013 and 2014.

In 2011, all tags were deployed with the factory-fitted wire tether. From 2012 onwards, tags were deployed with a 550 lb breaking strain Dyneema tether tied directly to the tag and dermal anchor. Knots were sealed with heat and strong adhesive. Tether length was set at 10 cm for PATs (MK10 and MiniPAT). After trials of various lengths between 80 and 150 cm, towed tags were deployed with a set length of 120 cm (SPOT5 and MK10F). All tag floats were painted with copper-based blue antifouling paint to deter growth of epibionts (such as barnacles and other fouling invertebrates) and to minimise predation attempts on the tags.

### Pop-up archival satellite tags

Ten MK10 PATs were deployed; seven in 2011, two in 2012 and one in 2014 (Table 1). Mean tag retention time for MK10 tags was 67 days ± 19 (range 10–120; median = 73; n = 7). Mean tag retention for MiniPATs was 175 days ± 5 (range 137–180; median = 180; n = 8). Tag retention for MK10 tags was significantly less than in MiniPATs (Mann-Whitney U test, P < 0.05) and their failure rate was also higher: 30% compared to 100% success rate for MiniPATs programmed for less than 180 days. MK10 tags were set for deployment periods of 120–365 days with just two MK10s lasting for a full 120-day set deployment.

MiniPATs collected light-level data which were used to determine location. Histogram bin data on temperature and depth as well as temperature and depth time series data were also collected. The 2012 models had an 8 GB memory card and time series data could only be collected on either depth or temperature due to the small memory space. For this study, depth was given priority. The 2014 MiniPAT models had a 16 GB memory card and collected time series data on both depth and temperature simultaneously. MiniPATs generally transmitted most of their archived data (mean 70%) via Argos before their batteries were exhausted.

Ten MiniPATs were deployed, five in 2012 and five in 2014 (Table 1). Two tags set for long deployment durations of 365 days failed to report. All other tags were set for a deployment of 180 days, and all transmitted data. Of the eight tags that reported, only one detached before its intended pop-up date, 43 days early. No pop-up tags were successfully recovered throughout this study.

### Towed satellite tags

Twenty-eight SPOT5 deployments were made over 2013 and 2014 using 26 tags (Table 2). Two tags were recovered and re-deployed after detachment from the shark. Tag retention was lower than with the archival tags, ranging from 1 to 227 days with a mean retention time of 69 days. SPOT5 tags also recorded 12-hour histogram data for temperature collected within the previous 24 hours.

In 2012, we used five prototype tags (MK10-F) that collected Argos locations, light-level, depth, and temperature data, and possessed Fastloc Global Positioning System (GPS) capability. These tags provided fine-scale and accurate location information together with temperature and depth, and so for the first time combined behavioural data with accurate location estimates (depending on surface time and satellite coverage). This tag model collected both time-series data and binned histogram data for both temperature and depth. These tags are designed to send opportunistic transmissions of data throughout their deployment. However, the initial design of the tag (used in 2012) was insufficiently buoyant to reach the surface when attached to a swimming shark. Small floats, added to the tether to aid buoyancy, may have resulted in reduced attachment time due to increased hydrodynamic resistance. All five tags were set for a 180-day deployment, but two failed to report and one had a short deployment of only 17 days. The remaining two tags made a full deployment (Table 3). Few location data were received from the tags, presumably due to the flawed design.

The MK10Fs we used in 2013 were re-designed into a ‘SPOT 5’ style tag, which had improved buoyancy and a longer mean deployment duration of 87 days (54–129 days). The tags were re-designed again in 2014. Two of the new style tags were deployed, but retention time was 49 days for both tags (respectively).

Location data were split into two categories for further analyses: (1) light-level based locations from archival tags, and (2) Argos locations from towed tags.

### Light-level analysis

All data were transmitted and collected through the Argos system and downloaded via their website www.argos-system.cls.fr. The Wildlife Computers’ DAP 3 processor was used to best estimate the location of the sharks equipped with archival tags. The DAP 3 processor uses a Hidden Markov Model with the forward and backward algorithm at a 0.25° grid size with light levels, sea surface temperature (SST), and any applicable Argos or fastloc positions, as well as deployment and pop-off locations, used to estimate location and generate a surrounding confidence area. Most-likely locations were determined using a spline interpolation. Archival tags provided locations calculated from light-level data. Light-level locations had a mean error radius of ~50 km (Wildlife Computers, 2015).

### Argos location analysis

Tags fitted with an Argos transmitter (SPOT and MK10F) used standard Doppler-based geo-location to track the position of the shark. An accuracy estimate was assigned and a location class was provided (A, B, 0, 1, 2, 3). Class A and B had no error estimates, whilst classes 0, 1, 2 and 3 had an estimated accuracy of >1500 m, >1000 m, >500 m, >150 m, respectively. To facilitate regular data downloads from the Argos system, accounts were set up through Seaturtle.org’s Satellite Tracking and Analysis Tool (STAT). STAT automatically downloads Argos data daily and stores it online. To further improve location data, the Douglas filter was applied in Movebank (http://www.movebank.org). This tool is based on a Maximum Redundant Distance (MRD) filter and removes unrealistic locations. Argos locations with a B location class or better were included in analyses and an MRD radius of 100 km was set. One location per day was used for all further analyses by employing the “Best of Day filter” in Movebank.

### Temperature & depth

Depth and temperature data collected by satellite tags were analysed to investigate behaviour and habitat preference in relation to season and location. As the Arabian Gulf is shallow throughout with a maximum of just over 90 m depth, we defined “relatively deep” dives as > 40 m depth when discussing depth in the Gulf. Outside the Gulf, we define “deep dives” as > 100 m depth. SPOT tags were only able to collect temperature data and not depth data. Otherwise, all tags were programmed to collect data from 6 am to 6 pm (day time) and 6 pm to 6 am (night time). Time-at-temperature (%) was summarised into 11 temperature bins (Table 4) and time-at-depth (%) presented as seven depth bins (Table 4). Diel differences in temperature and depth were compared for tags capable of collecting depth data (MK10, MiniPAT & MK10F).

**Table 4:** Temperature and depth bins used throughout the satellite tagging study.

	Satellite Tag Bin Range										
	1	2	3	4	5	6	7	8	9	10	11
**Temp (°C)**	0–12	12–15	15–18	18–21	21–24	24–27	27–30	30–33	33–36	36–39	39+
**Depth (m)**	0–2	2–10	10–20	20–50	20–100	100–400	400+	NA	NA	NA	NA

A paired-samples t-test was conducted to compare the mean depth at four time intervals throughout the day (00:00–06:00, 06:00–12:00, 12:00–18:00, 18:00–00:00) within all sharks tagged with an MK10F satellite tag both whilst located within and whilst outside of the Al Shaheen area.

Tags that provided time-series data (n = 31) were used to investigate individual movement and behaviour of whale sharks throughout the tag’s deployment. Tags capable of time-series data collection were programmed to take temperature and depth measurements at 10-minute intervals for the entire deployment. To investigate circadian behaviour in more detail, hourly mean depth data were also split between the data obtained within whale shark aggregation area at Al Shaheen during the tuna spawning season (May-Oct) and those obtained outside the tuna spawning season (Nov-Apr).

### Determining tag detachment

Temperature and depth data were used to determine the time at which the tag detached from the shark, similar to the methodology described in Hearn et al [28]. For tags collecting and transmitting time series data the detachment time could be determined shortly after the tag floated to the surface as it then maintained a uniform depth and a constant temperature matching the local sea surface temperature (SST). For tags collecting and transmitting only histogram data, the point of detachment was determined from the tag recording data from within a single ±3°C temperature bin, within which was the local SST recording at the time, for at least three days. After three days of static temperature recording, the time at which the tag changed to this behaviour was chosen as the detachment time. The SPOT tags used in this study were capable of storing collected data messages in the buffer for a period of days. These stored data messages were then transmitted at a later time when the tag reached the surface.

### Kernel density analysis

All transmitted locations were input to ArcGIS 10.2.1. The “kernel density tool” was used to calculate occurrence magnitude per km^2^. The Minimum Bounding Geometry (MBG), 50% and 95% Volume Contours (PVC) were produced to estimate areas of overall and core habitat usage. Both kernel density and PVC were produced following the methodology outlined in MacLeod [29]. Data were similarly split into summer (tuna spawning season; May-Oct) and winter (non-spawning season; Nov-Apr). Two separate kernel density analyses were produced, one for each season.

Data from four tags were selected as case studies to show important aspects of whale shark movements within and outside the Arabian Gulf and to illustrate the relationship between movements and environmental variables in more detail.

## Results

### Movements of whale sharks

#### Light-level locations

**Fig 2:**
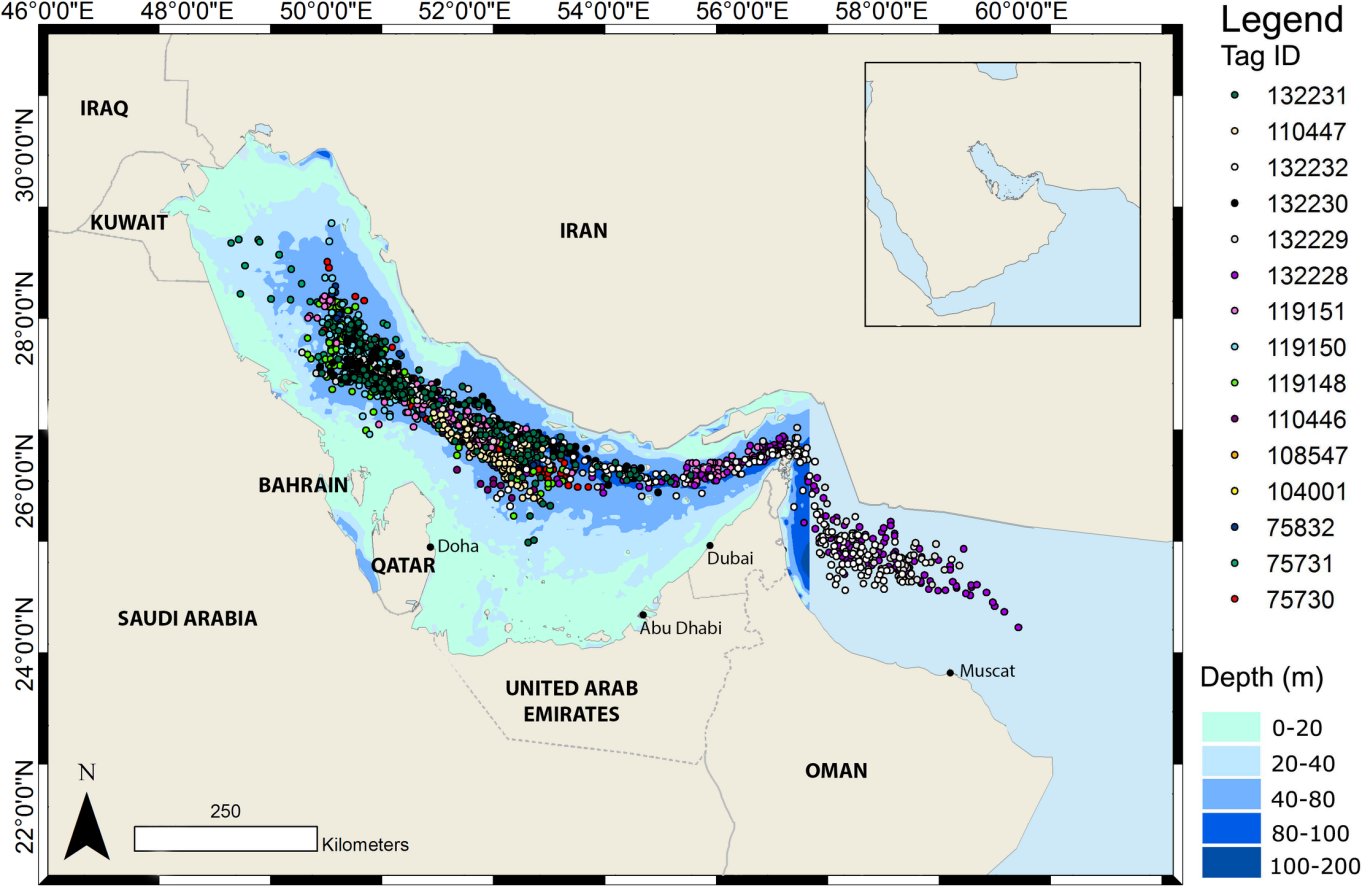
Overview of all locations generated from light-level analysis. The colour of the symbol indicates the tag ID as shown to the right.

Overall, locations derived from light levels showed dispersion throughout the relatively deeper waters of the Arabian Gulf as far north as Kuwait. Whale sharks rarely ventured into areas shallower than 40 m and no transmissions were made from water shallower than 20 m. Two sharks made a larger scale dispersal into the Gulf of Oman through the Strait of Hormuz (Fig 2).

#### Argos locations

**Fig 3:**
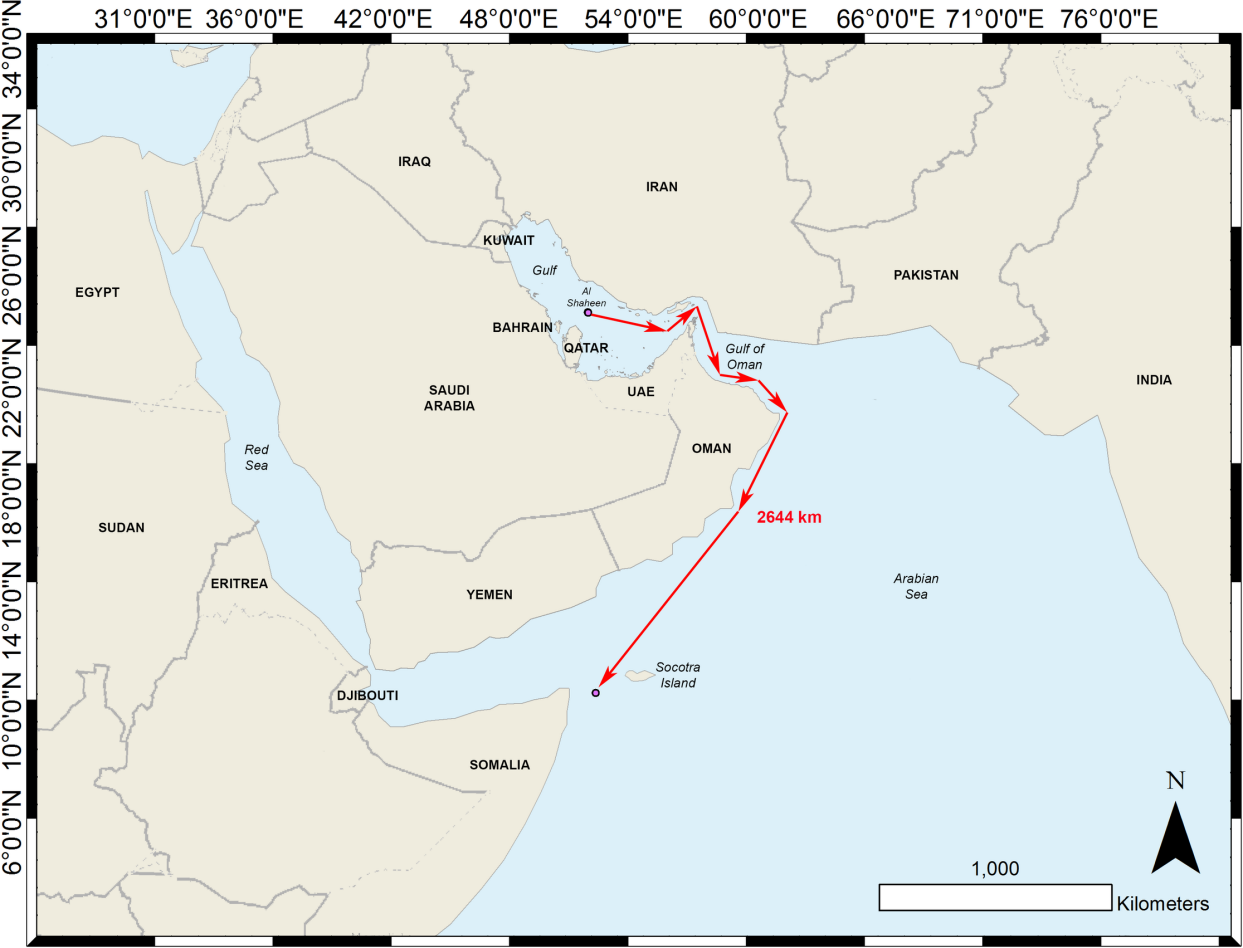
The estimated straight-line 2644 km journey of a presumed pregnant whale shark tagged in Al Shaheen.

All but one shark tagged with a GPS tag stayed within the Arabian Gulf and Gulf of Oman over the tag’s deployment. This large-scale horizontal movement was made by a presumably pregnant 9 m female, which was the only presumably pregnant female tagged in this study. This shark (qat14-023) left the study area and moved through Qatari, Iranian, UAE and Omani waters and the tag, detached in Yemeni waters approximately 35 km from the Somali maritime boundary and 36 km from the Island of Socotra, 37 days after tag deployment (Fig 3).

We investigated tag transmission data to eliminate the possibility that this tag was floating. We found that the tag transmission count did not increase between the deployment date of May 29 and June 29 2014. On June 29 2014, a single message was transmitted, indicating a brief surface event. Temperature histogram data were then transmitted for six days, during which time the tag moved through multiple temperature bins until our assigned detachment date of July 4 2014 when the tag started to report 100% time-at-temperature within a single temperature bin. The lack of transmissions from deployment to June 29, 2014, together with the temperature histogram data, show that the tag was still attached to the shark until the reported detachment date.

**Fig 4:**
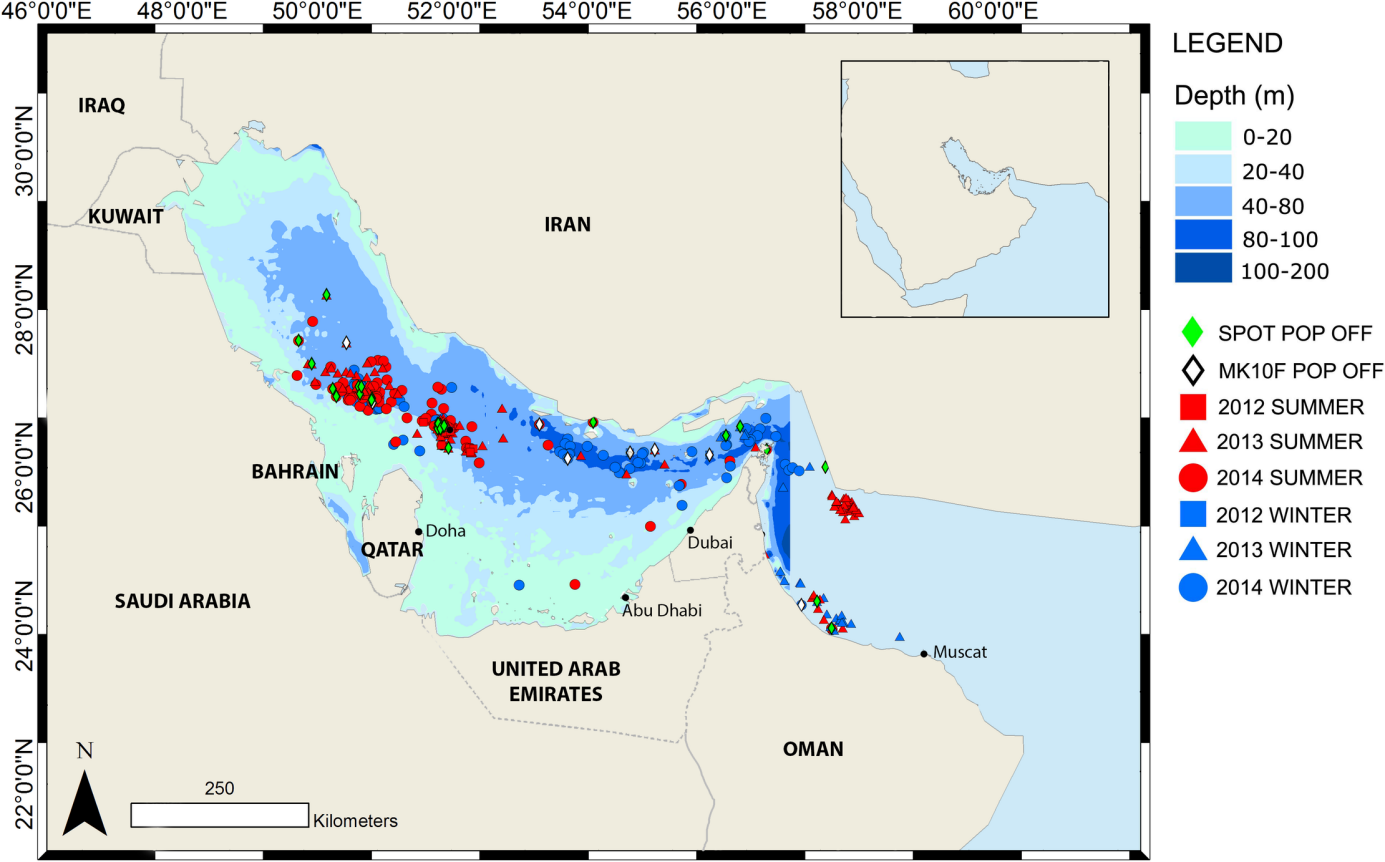
Overview of all locations transmitted by Argos tags from the Arabian Gulf and Gulf of Oman, split into years and season, together with Arabian Gulf bathymetry.

Otherwise the tagged whale sharks aggregated at Al Shaheen and off the Saudi Arabian Gulf coast during summer, but dispersed throughout the Gulf in winter. This pattern remained the same in all years of our study (Fig 4).

#### Kernel density analysis

**Fig 5:**
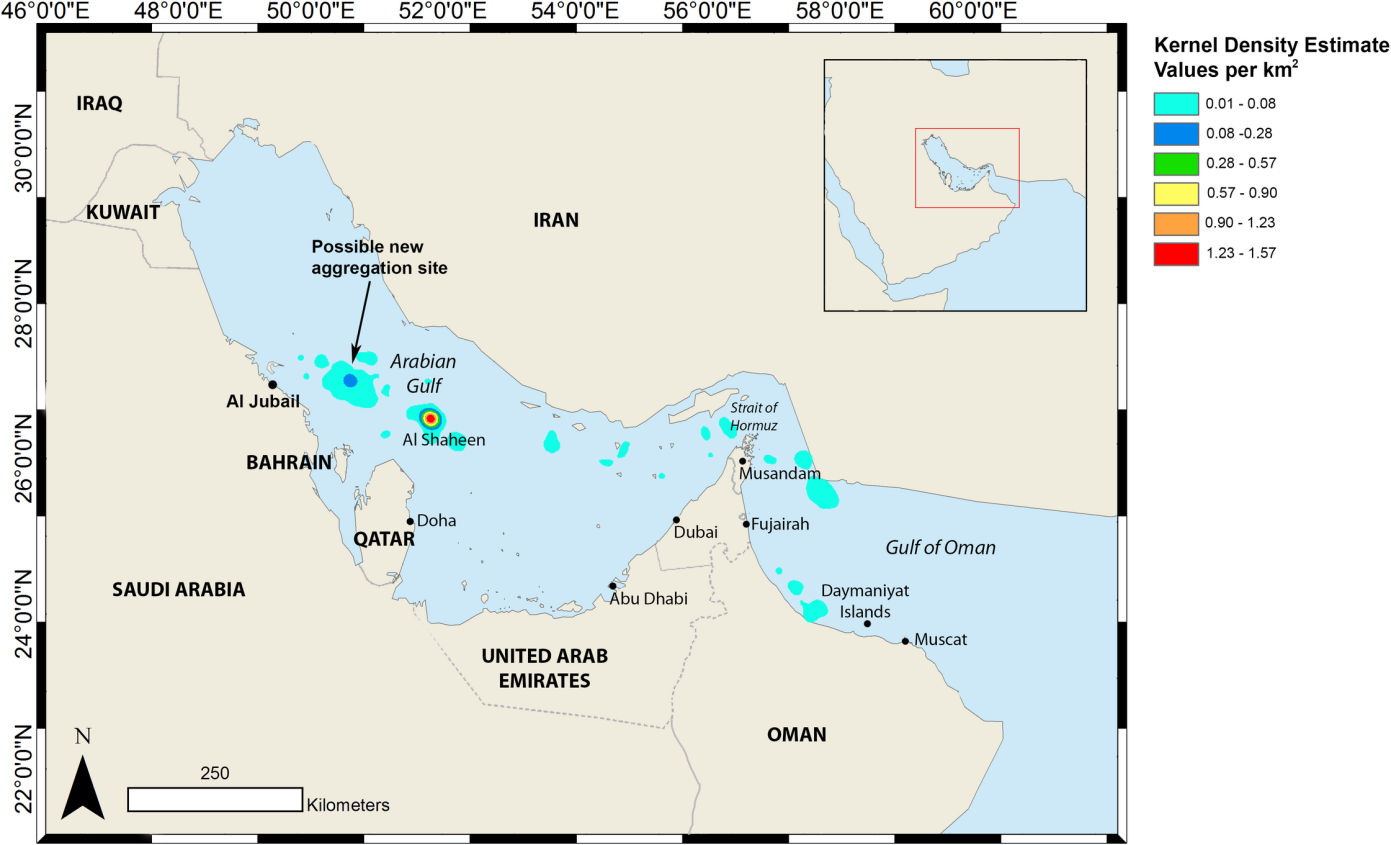
Kernel density analysis showing the pattern of transmission locations from satellite tags attached to whale sharks from the Al Shaheen area of Qatar and indicating a possible new aggregation site in Saudi Arabian waters.

Kernel analysis confirmed that Al Shaheen and the closely surrounding area is a highly significant area for tag transmissions (Fig 5). A possible new aggregation site for whale sharks was apparent in Saudi Arabian waters, 126 km north-west of Al Shaheen and 100 km offshore of Al Jubail.

#### Percentage volume contours and habitat usage

**Fig 6:**
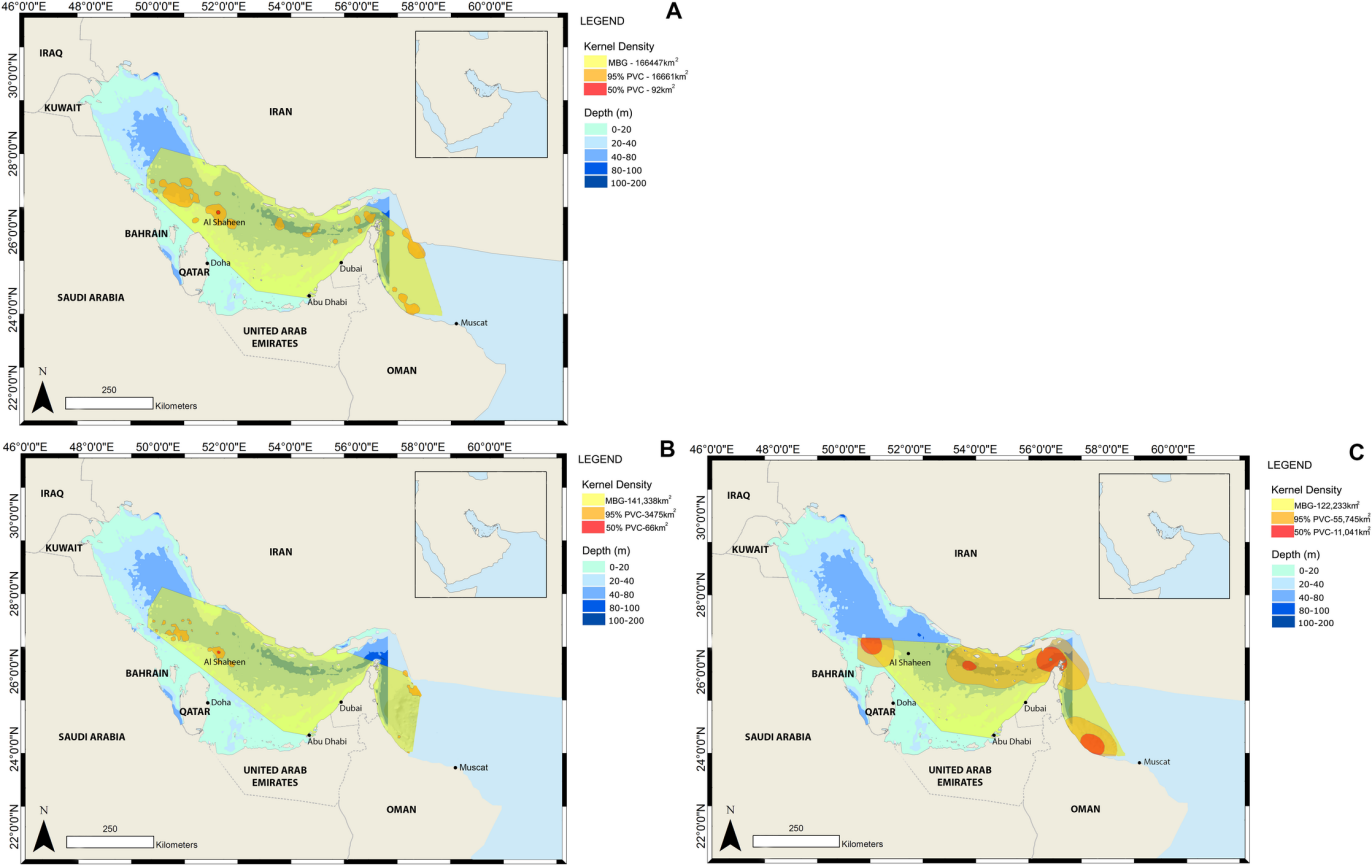
**Kernel Density Analysis and Minimum Bounding Geometry (MBG), showing the 50% and 95% Percentage Volume Contours (PVC) representing the core habitat use and total range respectively for: complete Argos locations (A), locations transmitted during summer (B), and locations transmitted during winter (C)**.

Kernel density analysis also identified the activity hotspot at Al Shaheen, as was seen in the raw location data. A minimum bounding geometry (MBG) for the locations in the Arabian Gulf and Gulf of Oman from all Argos transmissions was 166,447 km^2^. Core habitat (50% PVC) for the region was focused on Al Shaheen and encompassed a small area of 92 km^2^. The 95% PVC excluded outlying locations but included the same significant locations identified in the hotspot analysis plus a few additional areas which the sharks frequented (Fig 6).

Minimum Bounding Geometry (MBG) and PVC’s for summer were smaller than when data for summer and winter were combined. Although nine sharks left the Gulf during summer, the majority stayed until the end of the tuna spawning season.

The MBG and PVC’s increased in size during the winter, indicating that a larger area was being utilised, as sharks dispersed from summer aggregation areas. All winter core areas (95% PVC) were in locations deeper than 40 m showing that when the sharks disperse widely into the Gulf they still prefer habitat in excess of 40m which is restricted to the central and Northern side of the Gulf. Some sharks also moved to the Gulf of Oman via the Strait of Hormuz in winter (Fig 6).

**Fig 7:**
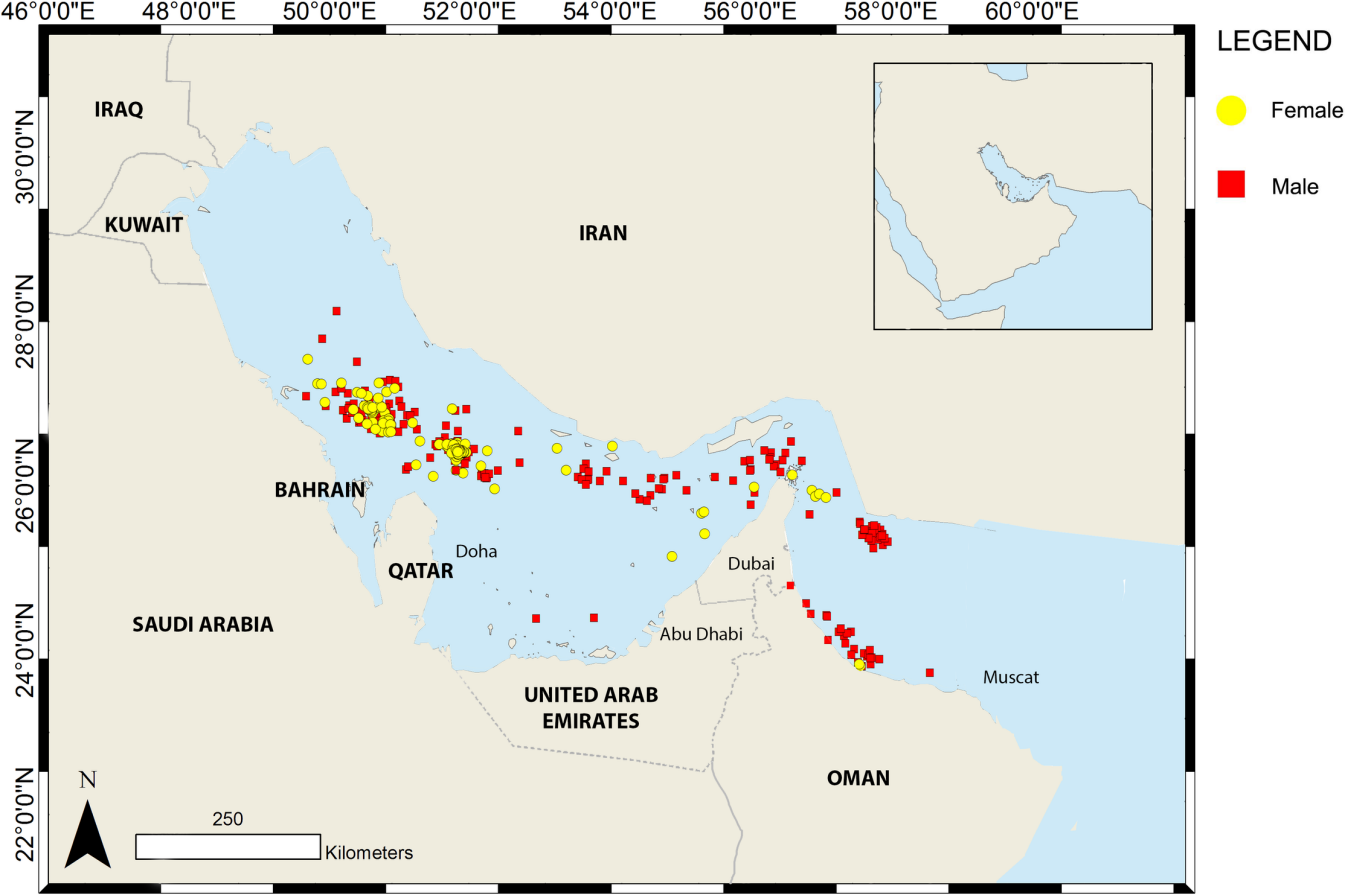
Overview of the Argos locations transmitted throughout the study period (excluding the Socotra pop-off point) for male and female whale sharks tagged in Al Shaheen.

There were no apparent differences in habitat preference between male and female whale sharks, or mature and immature animals. Both sexes aggregated off Al Shaheen or the Saudi site over summer before dispersing across the region throughout winter. Both sexes displayed the same affinity for the relatively deeper waters (>40 m) of the Arabian Gulf (Fig 7). The sole exception was the presumably pregnant female that swam towards Somalia (Fig 3); all other females stayed within the Arabian Gulf and Gulf of Oman area.

Whale sharks spent ~66% of time in temperatures between 24°C and 30°C (Table 5). Time spent in the four warmest temperature bins (24–27°C, 27–30°C, 30–33°C 33–36°C) was significantly higher during daytime, with sharks moving into cooler water at night (X^2^ = 121.692; p<0.05).

**Table 5:** Percentage time-at-temperature for 50 whale sharks tagged with temperature recording satellite tags at Al Shaheen between 2011–2014.

	Temperature Bins							
	18–21	21–24	24–27	27–30	30–33	33–36	36–39	39+
**Day (%)**	2	10	29	30	18	10	2	<1
**Night (%)**	<1	4	28	47	16	4	<1	0
**Overall (%)**	<1	11	29	37	16	6	<1	<1

Whale sharks spent ~79% of their time above 50 m (Table 6). Time spent in the five most frequented depth bins (0–2 m, 2–10 m, 10–20 m, 20–50 m and 50–100 m) was significantly different between night and day, with sharks spending more time in deeper water at night (X^2^ = 46.402; p<0.05).

**Table 6:** Percentage time-at-depth for 31 whale sharks tagged with depth recording satellite tags at Al Shaheen between 2011–2014.

	Depth Bins (m)				
	0–2	2–10	10–20	20–50	50–100
**Day (%)**	24	8	9	30	28
**Night (%)**	11	10	16	41	21
**Overall (%)**	17	10	13	39	21

#### Diving behaviour

**Fig 8:**
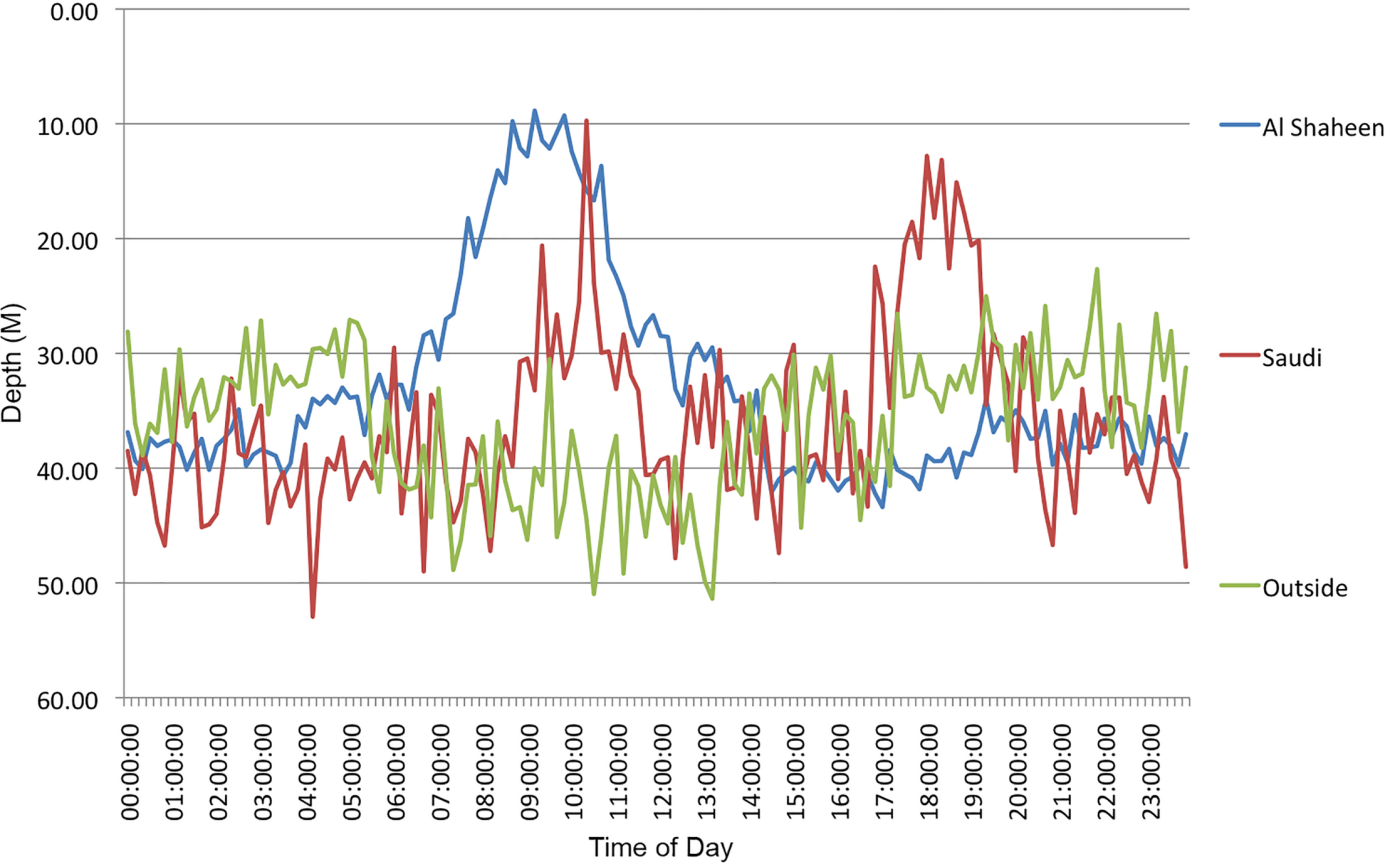
Mean actual hourly depths for time-series capable tags whilst sharks were within Al Shaheen (blue), outside of Al Shaheen (green) or within the possible aggregation site in Saudi Arabian waters (red).

A distinct diurnal pattern in behaviour of the whale sharks was evident whilst they were at Al Shaheen. They were shallower (mean = 20.6 +/- 7.9 m) from 6 am to 12 pm when at Al Shaheen in summer compared to when they dispersed over winter (41.8 +/- 4.4m; t = 13.7, p<0.0001). Sharks at the possible Saudi aggregation site showed a similar pattern of behaviour, but with a second movement to surface waters around dusk. Once the sharks were outside of either aggregation site, they spent most of their time between 30 and 50 m with no pronounced diel pattern (Fig 8).

#### Annual returns

Of the 55 satellite tagged sharks that were photo-identified, 32 (58%) returned to Al Shaheen in following years. Twenty-two returned the year following their tagging (Table 7), with two sharks returning even five years after they were tagged.

**Table 7:** Annual re-sighting data for the 32 identified and tagged whale sharks from the year they were first sighted.

Re-sights after first year of identification				
Year 1	Year 2	Year 3	Year 4	Year 5
22	16	10	6	2

Of the 32 sharks that returned to Al Shaheen, 22 (69%) were seen in one or more consecutive years. One shark returned to Al Shaheen in each of the five years of study (Table 8).

**Table 8:** Annual consecutive re-sights for the 32 whale sharks that returned to Al Shaheen.

Number of sharks re-sighted in consecutive years after first identification				
1 consecutive year	2 consecutive years	3 consecutive years	4 consecutive years	5 consecutive years
14	6	1	0	1

### Individual behaviour case studies

#### MiniPATs

**Fig 9:**
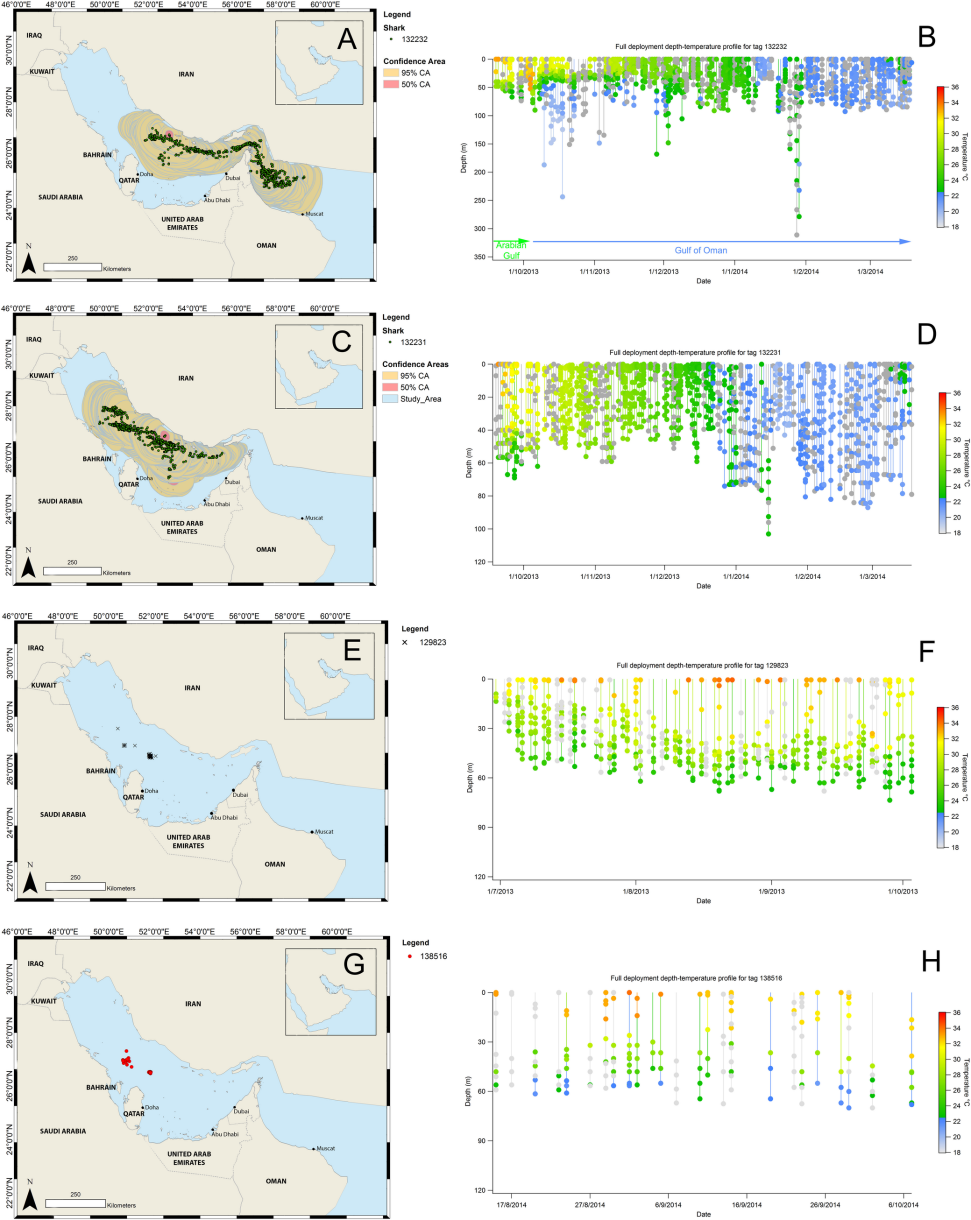
Estimated satellite tag locations produced from light level analysis throughout deployment of MiniPATs 132232 (A) & 132231 (B) including 95 and 50% location confidence areas and depth. Argos locations throughout deployment of MK10F 129823 (E) & 138516 (G). Deployment time-series depth-temperature data for MiniPATs 132232 (B) & 132231 (D) and MK10F 129823 (F) & 138516 (H). Grey spots represent where temperature data were unavailable.

MiniPAT 132232 was deployed for a full 180 days on shark qat13-098, a 7 m female. This shark left the Arabian Gulf through the Strait of Hormuz, indicated by an abrupt temperature change, and spent the remainder of the deployment within the Gulf of Oman. This shark made the deepest dive of any within this study, to a depth of just over 300 m (Fig 9A and 9B).

MiniPAT 132231 made a full 180-day deployment on shark qat13-095, a 7 m male. This shark experienced both the warmer summer and colder winter temperatures of the Arabian Gulf. A distinct cooling was recorded as the region transitioned from summer to winter. Light-level locations suggested that the shark stayed within the Arabian Gulf, however, the depth-temperature profile showed that the shark dived to more than 100 m, which means that it must have entered the Strait of Hormuz (Fig 9C and 9D) but did not move into the Gulf of Oman.

#### MK10Fs

MK10F 129823 made a 96-day deployment on an 8 m male shark, qat11-097. Argos locations showed that the shark did not leave the Arabian Gulf during the deployment. The time-series data show the surface waters of the Arabian Gulf started to cool around the beginning of October following peak temperatures in August and September. Water temperature was stable throughout the summer months with a temperature of around 34°C. This shark spent most of this time within Al Shaheen before moving north towards the possible Saudi Arabian aggregation site prior to the tag detaching in October (Fig 9E and 9F).

MK10F 138516 made a 54-day deployment on a 6 m male shark, qat14-041. This shark spent time in Al Shaheen and then moved to the possible aggregation area in Saudi Arabia where the tag popped off. Time-series data transmission displayed standard depth and temperature data for the central Arabian Gulf (Fig 9G and 9H)

## Discussion

Almost all the whale sharks tagged at Al Shaheen spent extended periods (several months) inside the Arabian Gulf, where surface temperatures can be greater than 35°C and the maximum depth is just over 90 m. Whale sharks tolerated high surface temperatures for several hours daily over that period. These same sharks were then seasonally exposed to mixed and cool waters over winter, with one shark exposed to temperatures constantly below 22°C for over a month (Fig 9D), demonstrating that individuals face a broad range of temperatures. The extreme environment of the Arabian Gulf presents an opportunity to learn more about how behavioural thermoregulation may allow whale sharks to maintain a reasonably even body temperature range, and what the optimal temperature envelope is likely to be for this wide-ranging species.

### Residency and dispersal from Al Shaheen

Kernel density analysis identified Al Shaheen as the core area utilized by tagged sharks during the tuna spawning season of May through to September [23]. An area of 66 km^2^ was identified as core habitat. Some individual whale sharks display a high affinity for Al Shaheen through the summer, apparently spending the whole tuna spawning season at this site. Affinity to Al Shaheen throughout this spawning season suggests that the production of eggs may be sufficient to support the dietary needs of sharks for several months. Hoffmayer et al. [30] described the feeding behavior of whale sharks in the Gulf of Mexico that were feeding on tuna spawn from the little tunny, *Euthynnus alletteratus* (Rafinesque, 1810) and estimated the consumption of eggs at 9000 m^2^ water filtered. Heyman et al. [31] described an aggregation site in Belize where whale sharks feed on snapper spawn, they report that fish eggs have a high caloric content, and when highly concentrated, such as at Al Shaheen, become an important food source. Tyminski et al. [9] calculated that a 6.2 m whale shark feeding on tuna spawn in the Gulf of Mexico for 11 hours, in similar conditions to Al Shaheen, would ingest 6 to 10 times the calories that an equivalent shark in captivity is rationed per day. Tuna spawn is likely to be an extremely efficient food source and this may explain the high affinity of some individuals for Al Shaheen.

While some sharks did transit through the Al Shaheen site relatively quickly, only spending a few days at the site, evidence from the kernel density analysis in this study suggests that additional aggregation sites exist elsewhere in the Gulf, specifically offshore of Al Jubail in Saudi Arabian waters. Several males and females of varying lengths visited this area. The location has a similar bathymetry to the Al Shaheen site and could also be a seasonal feeding area for whale sharks, although the evidence is presently circumstantial.

During the winter months the MBG and PVC increased in area as sharks dispersed throughout the Arabian Gulf and Gulf of Oman. Argos locations showed that the sharks rarely moved north past Saudi Arabian waters or towards the shallower waters close to the coast of Qatar and the UAE. Whale sharks have been reported from these areas but these sharks are usually juveniles less than 4 m in length and sightings usually occur in the winter months when the waters are cooler [6]. Sharks have also been reported from Kuwait [6]; these sharks were also juveniles less than 6 m in length. Only one of 58 tagged sharks, a presumably pregnant female, left this region over the whole tagging period from April 2011 to the end of 2014. Robinson et al. [6] used individual spot patterns of whale sharks to track their movements around the Arabian region and found that although sharks moved in and out of the Arabian Gulf through the Strait of Hormuz, as similarly documented in this study, none of the individuals encountered in either Gulf have been sighted outside of this area. Tag transmissions reduced in frequency once the sharks left Al Shaheen, suggesting that they modified their feeding behaviour. This made their journeys difficult to track using towed tags. Although PAT tags have a large factor of error in their light intensity-based locations, larger-scale movements away from the study area should nevertheless have been detected. The amount of time the sharks spent in these restricted waters highlights the importance of the Arabian Gulf as whale shark habitat.

### Influence of life stage on movements

Whale sharks recorded during this study ranged between 4 and 10 m in length. Given that whale sharks are born at around 50–60 cm and may grow to 18–20 m [3], the absence of small juveniles or large adult sharks suggests that this region is utilised solely by larger juveniles and smaller mature sharks [6]. Whale sharks of <3 m or >10 m are rarely reported anywhere in the world, so it is likely that these life stages either inhabit offshore waters or rarely approach the surface where they are obvious to human observers. There were no clear differences in the movements of juvenile versus adult sharks, excepting the single presumably pregnant female. Both sexes aggregated in the summer and dispersed in the winter, using the same stretch of relatively deeper water in the Arabian Gulf. Both sexes traveled through the Strait of Hormuz into the Gulf of Oman and both also utilised the possible Saudi aggregation site.

Few mature males have been previously tagged elsewhere, and to our knowledge this study reports on the first presumably pregnant female whale shark tagged in the Indian Ocean basin. This singular female was 9 m TL and tracked with a SPOT5 tag. The shark remained in Al Shaheen for a couple of days after tagging and then headed straight out of the Arabian Gulf. The tag detached close to the Socotra Islands (Yemen) after a 37-day journey during which no location signal was transmitted, although a single message was received on June 29^th^, indicating a brief surfacing event. The few mature female whale sharks tracked previously, largely in the Eastern Pacific [27,32,33] have shown a clear preference for oceanic habitat. A 7.5 m female with a “noticeable enlarged” pelvic area was tagged in the Gulf of Mexico and also showed a preference for an oceanic habitat [7]. The significance of this movement is unclear without a larger sample size, but targeted tracking of this life stage is certainly of interest with regards to reproductive ecology. Whale sharks of >9 m length in this study were rarely successfully tagged as the skin on such large sharks is extremely difficult to penetrate with the dermal anchor. Alternative tag deployment or attachment techniques are under investigation.

### Depth and temperature

Sharks within the Arabian Gulf have limited access to waters in excess of 90 m [34]. Transmission locations and bathymetry data showed that most transmissions were from areas with bottom depth between 40 and 60 m, corresponding to the depth of the majority of the central Arabian Gulf ridge. A preference for relatively deeper water was observed in areas along the Iranian coastline during the winter months after the sharks had dispersed from Al Shaheen. Transmissions from locations in deeper water occurred after sharks dispersed through the Strait of Hormuz and into the Gulf of Oman, where waters are deeper than 90 m, although even in the Gulf of Oman the transmission locations indicate that sharks rarely surface while in waters in excess of 100 m depth. Only one shark made a dive in excess of 300 m (Fig 9B) and dives of >100 m were uncommon. This is in contrast with many other satellite tagging studies that have shown frequent and deep dives to almost 2000 m [7,9,22,35]. Deep diving in whale sharks has been linked to feeding [16], energy saving while travelling [36] or thermoregulation [21]. The lack of deep diving in our overall study is almost certainly due to the shallow waters (<90m) found in the Arabian Gulf. The lack of deep dives where sharks had access to deeper water (>100m) while in the Gulf of Oman may perhaps be because of high prey availability in shallow waters in the region, negating the need for deep foraging dives. Similarly, Rowat & Gore [37] found that sharks frequenting waters of the Seychelles spent 96% of their time in waters less than 100 m depth, and Graham et al. [35] described whale sharks as epipelagic inhabiting waters between 50 and 250 m deep. Berumen et al. [5] report that sharks in the Red Sea frequent waters less than 50 m depth. At the same time the whale sharks tagged in Al Shaheen displayed a distinct preference for the relatively deeper waters of the Arabian Gulf.

Surface water temperatures in the summer within the Arabian Gulf are frequently in excess of 35°C [38]. Water temperature in the top 10 m at Al Shaheen can also exceed 35°C [23]. However, the relatively deeper central ridge of the Arabian Gulf forms distinct temperature layers. During August sharks at Al Shaheen feed at the surface for >6 hours a day, but at 60 m depth water temperatures are comparatively cool at 18°C (S. Bach, unpubl. data) even during summer. This temperature layering can be clearly seen in all time-series, depth and temperature data from this study (Fig 9). Although we did not specifically examine this here, it is possible that access to cooler waters is a requirement for short-term foraging in extremely hot water. In tropical Mexican waters, where whale sharks feed similarly on tuna eggs near the surface, a clear diel pattern in depth use (as also observed at Al Shaheen) was hypothesised to relate to either heat dissipation or overnight post-feeding thermotaxis to improve digestive uptake [9]. The water column in the Gulf starts to mix in mid-October. After mixing, temperatures hold in the low-20’s throughout the water column. One shark was observed within the Gulf for the full deployment of the tag and over the winter period (Fig 9D). Although the Gulf is warm in the summer, this shark was exposed to water temperatures below 22°C for more than one month in the winter months. This area provides an ideal “natural experiment” for further investigation of the influence of temperature on whale shark movement ecology.

### Re-sights and migrations

The Convention on the Conservation of Migratory Species (CMS) defines a migratory species as ‘the entire population or any geographically separate part of the population of any species or lower taxon of wild animals, a significant proportion of whose members cyclically and predictably cross one or more national jurisdictional boundaries”. Rowat & Brooks [3] report that whale sharks are highly mobile and concluded that there is no rigorous evidence from satellite tagging studies to show that whale sharks disperse from an area of tagging and then return sometime later, displaying true annual migration. However, Hueter et al. [7] stated that the return of individuals to aggregation sites was common, and reports a shark seen in six consecutive years. Hearn et al. [28,32] also describe the return of satellite-tagged sharks to the site of tagging after a large-distance oceanic dispersal. Within this study, 59 sharks were satellite tagged and 55 of those had identifiable spot pattern images recorded. Of those 55 sharks, 32 returned to Al Shaheen in at least one further year after first being recorded and all sharks that still had tags attached at the end of the spawning season crossed national jurisdictions into the territorial waters of other countries including Saudi Arabia, Iran, The United Arab Emirates, and Oman. Our re-sight data shows that a high proportion of whale sharks make annual migrations to Al Shaheen, after which they disperse into the wider Arabian Gulf and Gulf of Oman.

## Concluding remarks

The Arabian Gulf is, during the summer months, the warmest sea in the world, yet Al Shaheen hosts one of the largest-known whale shark aggregations. Here we have shown that over winter, despite a precipitous drop in temperature, the majority of satellite-tagged sharks remained in the region. Individual whale sharks were shown to migrate repeatedly to Al Shaheen across years. We have identified a likely second aggregation site in the Gulf, off Al Jubail in Saudi Arabia, which should be investigated further. Whale sharks are only afforded species-specific protection within the UAE amongst GCC countries, although all sharks species are protected from fishing in waters of Saudi Arabia and Kuwait. Following the end of the tuna spawning season, every tracked shark in this study dispersed across national boundaries, with many moving through multiple jurisdictions. The protection afforded to them through existing conservation legislation may thus be relatively limited. There is a need for regional and international collaboration to determine how best to protect the species within the region.

## Supporting information

S1 Fig An image of whale shark taken at Al Shaheen. (TIF)

S2 Fig An image of a researcher satellite tagging a whale shark taken at Al Shaheen. (TIF)

S1 Data The satellite transmitted data for each whale shark deployed tag included in this study. (XLSX)
