# Supplementary figures and images for: Some like it hot: Repeat migration and residency of whale sharks within an extreme natural environment
Source: PLoS One. 2017 Sep 21;12(9):e0185360. doi: 10.1371/journal.pone.0185360 (PMC5608409; doi:10.1371/journal.pone.0185360)

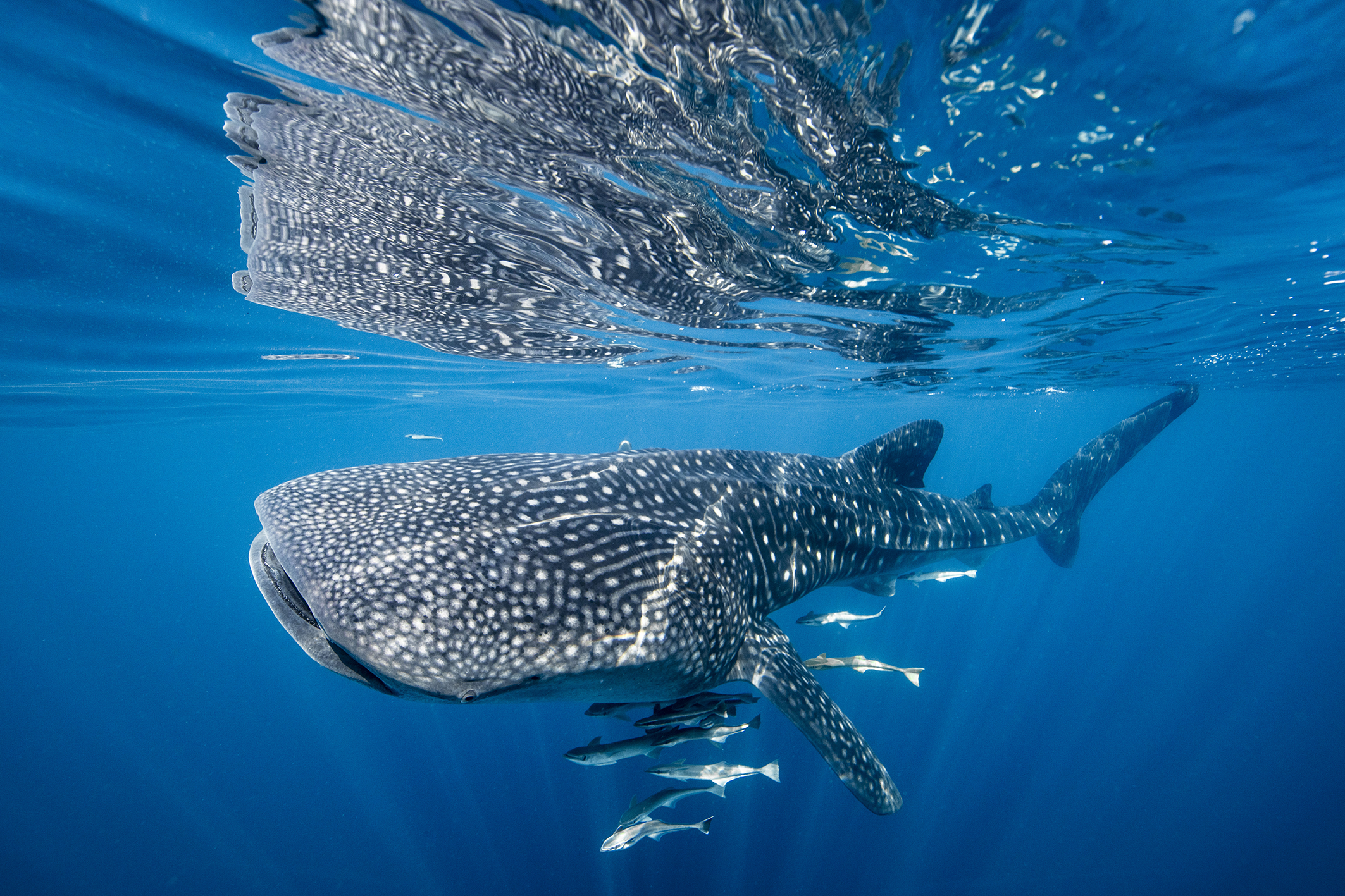

Supplement: S1 Fig — (TIF) [file pone.0185360.s001.tif]

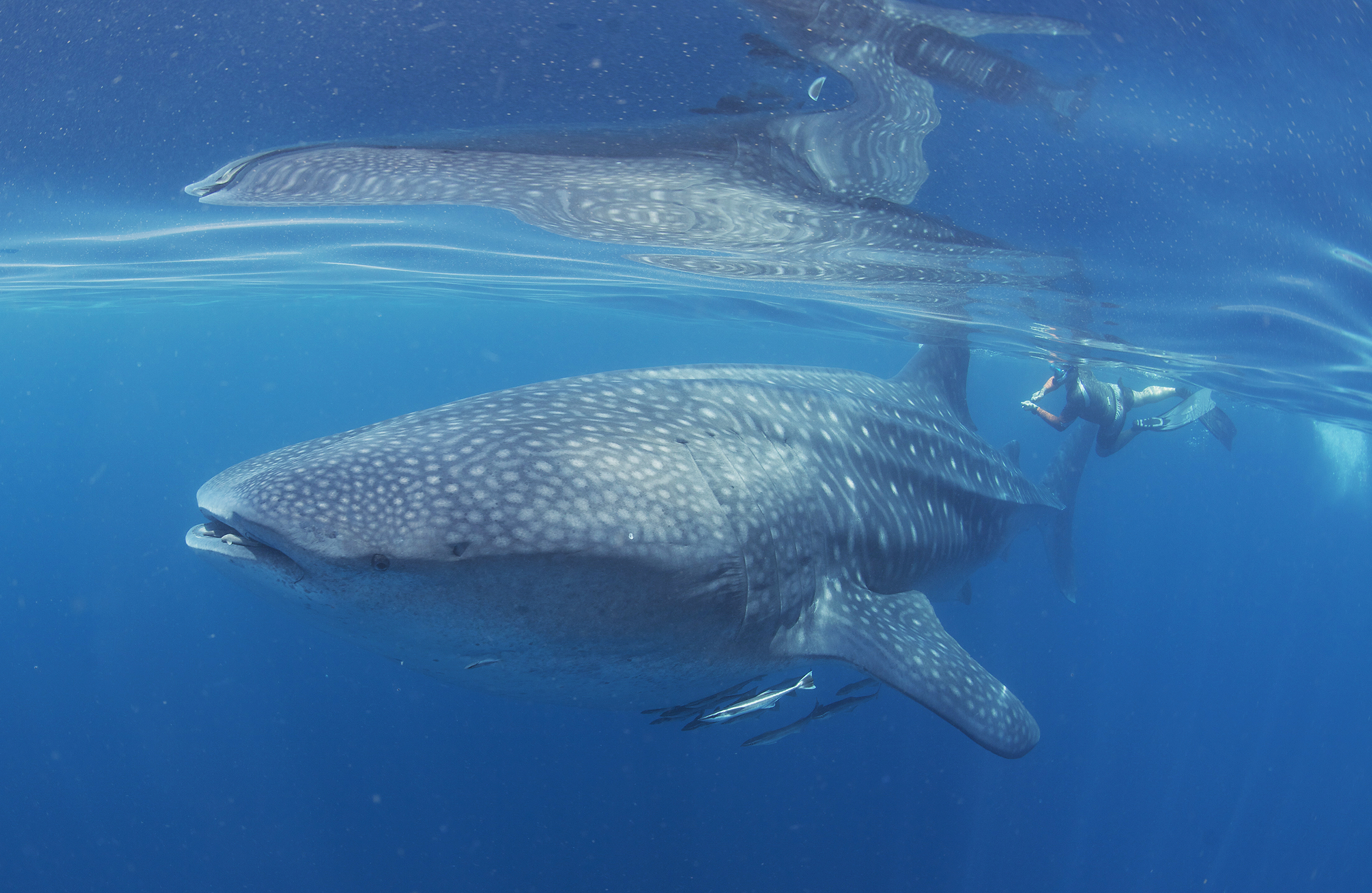

Supplement: S2 Fig — (TIF) [file pone.0185360.s002.tif]
